# Centralized Unmanned Aerial Vehicle Mesh Network Placement Scheme: A Multi-Objective Evolutionary Algorithm Approach

**DOI:** 10.3390/s18124387

**Published:** 2018-12-11

**Authors:** Sérgio Sabino, Nuno Horta, António Grilo

**Affiliations:** 1 Instituto Superior Técnico-Universidade de Lisboa, Av. Rovisco Pais 1, 1049-001 Lisboa, Portugal; nuno.horta@lx.it.pt (N.H.); antonio.grilo@inov.pt (A.G.); 2 INESC-ID, R. Alves Redol 9, CP 1000-100 Lisboa, Portugal; 3 Instituto de Telecomunicações, Av. Rovisco Pais 1, 1049-001 Lisboa, Portugal

**Keywords:** unmanned aerial vehicles, genetic algorithm, mesh networks, optimization, MOEA, NSGA-II

## Abstract

In the past, Unmanned Aerial Vehicles (UAVs) were mostly used in military operations to prevent pilot losses. Nowadays, the fast technological evolution has enabled the production of a class of cost-effective UAVs that can service a plethora of public and civilian applications, especially when configured to work cooperatively to accomplish a task. However, designing a communication network among the UAVs is a challenging task. In this article, we propose a centralized UAV placement strategy, where UAVs are used as flying access points forming a mesh network, providing connectivity to ground nodes deployed in a target area. The geographical placement of UAVs is optimized based on a Multi-Objective Evolutionary Algorithm (MOEA). The goal of the proposed scheme is to cover all ground nodes using a minimum number of UAVs, while maximizing the fulfillment of their data rate requirements. The UAVs can employ different data rates depending on the channel conditions, which are expressed by the Signal-to-Noise-Ratio (SNR). In this work, the elitist Non-Dominated Sorting Genetic Algorithm II (NSGA-II) is used to find a set of optimal positions to place UAVs, given the positions of the ground nodes. We evaluate the trade-off between the number of UAVs used to cover the target area and the data rate requirement of the ground nodes. Simulation results show that the proposed algorithm can optimize the UAV placement given the requirement and the positions of the ground nodes in the geographical area.

## 1. Introduction

Unmanned Aerial Vehicles (UAVs), also known as drones, refer to aircraft with no human pilot on board. These are either programmed and fully autonomous or remotely and fully controlled from another location, e.g., ground or space station. There are various types of UAVs (e.g., fixed wing and multi-rotor), and they come in different sizes, raging from small (less than 5 kg) to large (over 4332 kg) [1]. Large UAVs are commonly used singly, for instance, in military operations such as border surveillance, strikes, and reconnaissance, whereas small UAVs may be utilized in swarms to accomplish a mission. With advancement in electronics and sensor technology, small UAVs are becoming massively present in many public and civilian applications, such as in search and rescue operations [2], aerial surveillance [3], tracking targets [4], agriculture field monitoring [5], network extension or compensation [6], and leisure, to mention a few.

The use of swarms of small UAVs has many advantages compared to a single and large UAV [7]. One of the key advantages is the cost to acquire and maintain small UAVs, which is generally much lower than the cost of a large UAV [8]. Swarms of UAVs can automatically reconfigure themselves in case of node failure or link break and accomplish the designated task. That is not possible with a single UAV. Additionally, when network coverage extension is needed, it may be easily achieved with swarms of UAVs by positioning additional UAVs in the target area and allowing them to operate through other already existing UAVs, unlike single UAV network coverage, which is limited by the communication range between the infrastructure and the UAV itself.

Although swarms of UAVs present many advantages, an important aspect to be considered when designing an application using multiple UAVs is the communication network, which poses many challenging issues, as described in [9,10]. Depending on the purpose of the application at hand, UAVs may be semi-stationary and hovering over the area of operations or move around at high speed, changing their relative positions. In the latter scenario, frequent topology changes are observed, which may lead to network partitioning and poor link quality. On the other hand, the commonly-used wireless ad-hoc network communication protocols or algorithms (e.g., proactive and reactive routing) cannot be directly used for UAVs [11]. For instance, since proactive routing protocols need to update the routing tables periodically, in the presence of a high degree of mobility and topology changes, it increases the number of control messages to be exchanged, which degrades the network performance. On the other hand, reactive protocols may introduce higher packet delivery delay each time they compute a new route to the destination node.

UAV placement schemes can help to mitigate the aforementioned issues by finding suitable positions to place UAVs while maintaining connectivity and improving the network performance. The UAV placement optimization schemes can be classified as centralized or distributed. The former assumes that the UAV positions are selected by a centralized entity and conveyed to the UAVs by means of a special purpose long-range low bit rate radio interface. On the other hand, in distributed approaches, UAVs work cooperatively to adjust their position based on local interactions to achieve optimal coverage. This work extends our previous work [12], where we considered the use of a swarm of UAVs as flying access points forming a mesh network among themselves, providing connectivity to ground nodes (GNs). Our main goal is to optimize the placement of the UAVs by choosing deployment positions for the UAVs in order to provide adequate wireless communication coverage to GNs in a target area, while fulfilling their Quality of Service (QoS) requirements.

This work is more related to centralized placement optimization. Given the nature of the problem requirements, we use Multi-Objective Evolutionary Algorithm (MOEA) techniques to optimize the UAV node placement considering the following objectives and constraints:

- Minimization of the number of UAVs needed to service the GN, while ensuring that the QoS requirements (here, measured as the physical data rate) are properly met.
- Minimization of the degree of dissatisfaction regarding the required data rate.
- The number of available UAVs is limited and must not be exceeded.
- The inter-UAV links do not necessarily employ the same technology as GN-UAV links. Inter-UAV links are considered in an abstract way, but constrained to a maximum range.
- It is assumed that the throughput values of the links between UAVs are high enough not to constrain end-to-end inter-GN traffic. Only GN-UAV links impose limits to the satisfaction of QoS requirements (end-to-end QoS shall be addressed in future work);
- GN-UAV links are orthogonal. This can be achieved, for example, by assigning different frequencies or orthogonal channel codes.

The main contribution of this work is to provide a meta-heuristic algorithm, which takes into account more than one objective and multiple constraints to best position the UAVs. Additionally, to reduce the search space and accelerate the convergence of the algorithm, we propose the use of convex hull algorithms to delimit the total area to be covered into a sub-area inside the convex hull formed by the 2D positions of the GNs as explained later in this paper. The remainder of this paper is structured as follows: Section 2 presents the related work. In Section 3, the system model is presented. Section 4 presents the problem definition and formulation as a Multi-Objective Optimization Problem (MOP). Section 5 presents our MOAEA implementation. The simulation results are presented in Section 6. Section 7 presents the simulation results’ discussion, and Section 8 concludes the paper.

## 2. Related Work

Optimal placement of UAVs has already been studied in the literature, whether considering single or multi-UAV scenarios. In [2], a single UAV was proposed for search and rescue application such as earthquake, flood, or bomb blast. The goal is to deploy a UAV to a position where it can bridge communication between two static nodes on the ground. It is assumed that the UAV hovers over the area in spiral or ladder search mode, sending hello/beacon messages at a regular interval. Upon receiving such a message, the GNs respond by sending their GPS positions back to the UAV. The UAV stores this information and continues hovering in the immediate surroundings to find a position based on the Received Signal Strength (RSS) and distance between the UAV and nodes on the ground. Simulation results showed that the algorithm provides maximum throughput and a low Bit Error Rate (BER) once the UAV is fixed at an optimal position. The drawback of this system is that it is only validated for two GNs. Therefore, as the number of GNs grows, the solution should consider energy constraints during the search process and bandwidth constraints when providing network access to GNs.

The authors in [13] have developed a framework named UAVNet. It is capable of autonomously deploying a wireless mesh network to interconnect two end systems using small quadrocopter-based UAVs with 802.11 s nodes on board. Each UAV would act as the access point and provide network access for regular IEEE 802.11 g wireless devices. There are two positioning modes to place the UAVs between the end systems. The first one is the location-based positioning mode. The latter uses the submitted GPS locations of the end systems and directs the UAV to the exact geographical position between these two GPS coordinates. The second one is the signal strength positioning mode. It extends the location positioning mode and includes also the received signal strength of the two end systems to calculate a more accurate position for the UAV. This takes the quality of the wireless link and other environmental factors into account.

Usually, the process of network densification in cellular networks uses fixed small cells (e.g., picocells and femtocells) to increase the network capacity based on the expected formation of hotspots. In places where temporary hotspots are formed, fixed small cells would remain under-utilized once the hotspots have moved to a different location or disappeared. The authors in [14] proposed small cells mounted on UAVs to offload User Equipment (UE) from the microcell infrastructure. The optimum placement points of the UAVs are determined using the K-means clustering algorithm. In their work, the performance metric was measured based on the RSS experienced by the UE. The simulation results have shown that as UAVs are able to position themselves in real time around the actual UE position rather than expected UE hotspots, they outperform equivalent small cell deployment.

In [15], Al-Hourani et al. presented a mathematical model that aims to optimize the flying altitude of UAV-based Base Stations (BSs) to maximize the coverage area on the ground. The Air-to-Ground (A2G) path loss model is based on their previous work [16]. The A2G path loss in [16] considered some parameters (αo, βo, and γo) that describe to a fair extent the general geometrical statistics of a certain urban area in which the Radio Frequency (RF) signal propagates. The authors refer to the additive loss incurred on top of the Free Space Path Loss (FSPL) as the excessive path loss (η), which has a Gaussian distribution. In the model, η is used to identify three different groups of GNs, namely G1, which is the group favoring the Line-of-Sight (LoS) condition; group G2 corresponds to GNs with No Line-of-Sight (NLoS), but still receiving coverage via strong reflection and diffraction; and lastly, group G3, which suffer from deep fading conditions. The probability of a certain propagation group (excluding G3) to occur at a certain elevation angle is computed. Differently from [16], in [15], η refers to the mean value of the excessive path loss rather than its random behavior. The authors were able to present a closed-form equation based on the elevation angle and the urban statistical parameters (αo, βo, γo).

In [17], Kalantari et al. proposed a 3D UAV placement scheme using the Particle Swarm Optimization (PSO) algorithm. Their system model envisioned the use of UAVs as flying base stations (referred to as drone-BSs) and limited their analysis to the downlink communications. The main goal of the proposed algorithm is to find the minimum number of drone-BSs and their 3D placement to service all GNs with some target QoS requirement. The A2G channel model is similar to the one presented by Al-Hourani et al. in [16], where the probability of LoS and NLoS connectivity between the drone-BSs and GNs was studied as explained previously.

Chen et al. [18] studied the optimum altitude to place a UAV when it is used to relay data from GN to a remote ground station for further processing. The new proposed model derives from the seminal A2G channel models [15,16]. Differently from the former models, the proposed model accounts for the hop from the UAV to the remote station. The study was limited to a single UAV, which in turn may limit the coverage area.

Mozaffari et al. [19] proposed a method to deploy multiple UAVs equipped with directional antennas. The aim was to maximize the coverage performance while ensuring that the coverage areas of UAVs do not overlap. The path loss model considered here is based on the model proposed in [16]. Although the A2G models and coverage schemes presented in [15,16,17,19] may be a good approximation in an urban environment, they do not address the coordination mechanism or connectivity among UAVs, i.e., the Air-to-Air (A2A) connectivity, which is important for network reliability. In another work, Mozaffari et al. [20] presented a study in which the main contribution was to analyze the coverage and rate performance of UAV-based wireless communication in the presence of underlying device-to device (D2D) communication links.

In [21], the authors presented a model for an optimal placement of UAVs to cover a set of targets, i.e., GNs. They considered two cost metrics, namely the number of UAVs and energy consumption, seeking to minimize both metrics. The authors assumed that each UAV had a minimum and maximum observation altitude. They also assured that the UAV’s energy consumption was related to this altitude, since the higher the altitude, the larger the observed area, but also the higher the energy consumption. The optimization problem was mathematically solved by defining an integer linear and a mixed non-linear optimization model.

The authors in [22] used the same assumption as in [21] to model an optimized UAV placement and formulate it as a multi-objective linear problem. The main difference is that, in [22], the connectivity among UAVs was considered as an additional constraint. In [22], the following objectives were to be minimized: the number of UAVs and the maximum flying altitude. Our work is closer to [22], though with some differences. Firstly, we consider using omni-directional antennas instead of directional. Secondly, one of our objectives is to minimize the difference between the assigned and required data rate, whereas one of their objectives is to maximize the flying altitude. Table 1 presents the main characteristics of the related work.

## 3. System Model

We consider a wireless network consisting of two kinds of nodes, GNs and UAVs, which are represented by the sets V and U, respectively. All nodes are assumed to be located in a rectangular area A with length $X_{max}$ and width $Y_{max}$. Nodes are equipped with omni-directional transceivers and a GPS. Therefore, they know their positions in the aforementioned rectangular area at any time. The position of a GN *v* is assumed to be on the ground with coordinates $q_{\left(x,y,0\right)}^{v}$, while the position of a UAV node *u* is represented in the 3D plane as $q_{\left(x,y,h\right)}^{u}$, where *h* is the flying altitude of *u*. We consider A as a suburban area and that the main factor affecting the service quality offered by a UAV is path loss. Furthermore, orthogonal channel codes are used to avoid interference between concurrent transmissions. Similar to [23], we employed the log-distance path loss model, where the received power is calculated according to the following expression:

(1) $$P_{r}\left(d\right)=P_{r}\left(d_{0}\right)-10\alpha \log_{10}\left(\frac{d}{d_{0}}\right)$$

where $P_{r}\left(.\right)$ is the received power at a given distance. In Equation (1), $d_{0}$ is the reference distance and α is the path loss exponent. UAVs are assumed to have the same operating characteristics, featuring the same transmit power, antenna gains, and enough energy storage to complete the mission, and they may fly at different altitudes. GNs can only communicate with each other through UAVs. Assuming communication between a GN and a UAV, *d* is computed as the Euclidean distance between their transceivers as follows:

(2) $$d=\sqrt{{\left(x_{u}-x_{v}\right)}^{2}+{\left(y_{u}-y_{v}\right)}^{2}+h_{u}^{2}}$$

**Figure 1:**
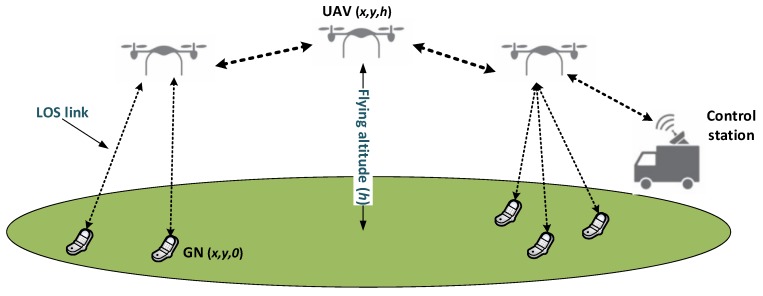
System model overview.

We define $D_{i}$ as the maximum achievable communication range for a given transmission mode *i* by simply manipulating Equation (1). $D_{i}$ is computed considering $P_{r}\left(d\right)$ as the receiver sensitivity at transmission mode *i*, and $P_{r}\left(d_{0}\right)$ is the received power at a reference distance $d_{0}$. The distance *d* between the transmitter and the receiver should not be greater than the maximum communication range $D_{i}$ for the required communication mode *i*. An overview of the proposed system is shown in Figure 1.

## 4. Problem Definition

Consider the network model presented in Section 3. The goal is to ensure that all GNs are covered and that the data rate requirements are met as much as possible when UAVs are used as relay nodes. We assume that there is a cost associated with each used UAV. Thus, minimizing the number of UAVs is desirable. On the other hand, GNs may have different data rate requirements. The satisfaction of data rate as GN requirements is closely dependent on the channel conditions (e.g., SNR), which also depends on the communication distance, which results from the number and placement of the serving UAV in the network. We intend to deploy as few connected UAVs as possible in suitable locations to enable communication between GNs, while satisfying multiple independent data rate requirements. In some instances, the QoS demands are competitive, i.e., one cannot satisfy them simultaneously. This gives rise to the need for finding solutions that try to balance them. This problem can be modeled meta-heuristically as a multi-objective optimization problem to find the trade-off among non-dominated solutions. In the rest of this section, we define the Multi-Objective Optimization Problem (MOP) and present the formulation of our UAV placement optimization problem as an MOP.

### 4.1. Multi-Objective Optimization Problem

A MOP can be stated as follows [24]:

(3) $$\begin{array}{ll} Minimize/Maximize\;f(\varepsilon ), & \;m=1,2,\ldots ,M\\ subject\;to:\;g_{j}\left(\varepsilon \right)\geq 0, & \;j=1,2,\ldots ,J\\ h_{k}\left(\varepsilon \right)=0, & \;k=1,2,\ldots ,K\\ \varepsilon_{i}^{\left(L\right)}\leq \varepsilon_{i}\leq \varepsilon_{i}^{\left(U\right)}, & \;n=1,2,\ldots ,n \end{array}\}$$

where *M* is the number of objective functions subject to *J* inequalities and *K* equality constraints. A solution ε is a vector of *n* decision variables, i.e., $\varepsilon ={\left(\varepsilon_{1},\varepsilon_{2},\ldots ,\varepsilon_{n}\right)}^{T}$. Each variable is subjected to a constraint called variable bounds. $\varepsilon_{i}^{\left(L\right)}$ represents the lower (*L*) bound, and $\varepsilon_{i}^{\left(U\right)}$ corresponds to the upper (*U*) bound. The set of all variable bounds defines the decision variable space Ω.

A solution ε that satisfies all constraints and variable bounds is named a feasible solution. The set of all feasible solutions is called the feasible region (or search space **S**).

**Definition** **1.** *Domination: A solution $\varepsilon^{\left(1\right)}$ is said to dominate the other solution $\varepsilon^{\left(2\right)}$, if the following conditions are verified:*

- *The solution $\varepsilon^{\left(1\right)}$ is no worse than $\varepsilon^{\left(2\right)}$ in all objectives, or $f_{m}\left(\varepsilon^{\left(1\right)}\right)$ is no worse than $f_{m}\left(\varepsilon^{\left(2\right)}\right)$ for all m=1,2,…,M;*
- *The solution $\varepsilon^{\left(1\right)}$ is strictly better than $\varepsilon^{\left(2\right)}$ in at least one objective, or $f_{\bar{m}}\left(\varepsilon^{\left(1\right)}\right)$ is better than $f_{\bar{m}}\left(\varepsilon^{\left(2\right)}\right)$ for at least one $\bar{m}=1,2,\ldots ,M$;*

**Definition** **2.** *Non-dominated set: Among a set of solutions P, the non-dominated set of solutions P’ is comprised of those that are not dominated by any member of the set P.*

**Definition** **3.** *Globally Pareto-optimal: This refers to the non-dominated set of the entire feasible space **S***.

### 4.2. Formulation of UAV Placement Optimization as an MOP

In this section, we formulate the problem in $R^{2}$ objective space. We seek to minimize the number of deployed UAVs and simultaneously minimize the difference between the data rate required by the GNs to transmit data and the data rates that result from the MOP solution.

#### 4.2.1. Minimize the Number of UAVs

**Figure 2:**
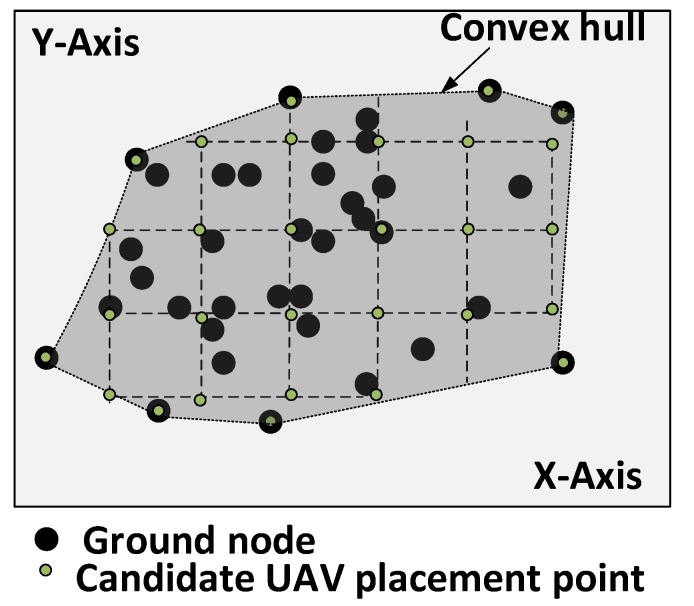
Convex hull formed by the GNs.

We start by identifying a set of potential UAV placement points *Q*, by finding a sub-area $a^{\prime }\subset A$, which corresponds to the area inside the convex hull (convex envelope) [25] formed by the GNs in A, as shown in Figure 2. We compute the convex hull to reduce the search space of the UAVs’ placement points in the target area. We intend to cover all GNs in $a^{\prime }$. Therefore, we discretize $a^{\prime }$ in a grid layout according to Equation (4).

(4) Δ=μD;μ∈[0,1]

where Δ is the distance between two neighboring UAVs, which is adjusted using μ. Let $q_{j}\in Q$ be the *j*th potential UAV placement point. We define $\delta_{q_{j}}^{u}$ as a binary variable to indicate which points are currently being used by a UAV as presented bellow.

$$\delta_{q_{j}}^{u}=\left\{\begin{array}{cc} 1 & \mathrm{if}\;\mathrm{UAV}\;u\;\mathrm{is}\;\mathrm{located}\;\mathrm{at}\;q_{j}\\ 0 & \mathrm{Otherwise} \end{array}\right.$$

We also define $\zeta_{v}^{u}$ as a binary variable to indicate which GNs are being serviced by each deployed UAV. It is assumed that a GN will be connected to the closest deployed UAV.

$$\zeta_{v}^{u}=\left\{\begin{array}{cc} 1 & \mathrm{if}\;v\;\mathrm{is}\;\mathrm{connected}\;\mathrm{to}\;\mathrm{UAV}\;u\\ 0 & \mathrm{Otherwise} \end{array}\right.$$

Our objective is to select points in *Q* such that:

(5) $$min\;\sum_{q_{j}\in Q}\sum_{u\in U}\delta_{q_{j}}^{u}$$

subject to:

(6) $$\sum_{q_{j}\in Q}\delta_{q_{j}}^{u}\leq 1,\forall u\in U$$

(7) $$\sum_{v\in V}\zeta_{v}^{u}\geq 1,\forall v\in V$$

Constraint (6) indicates that each UAV *u* cannot be placed in more than one point at the same time. Constraint (7) ensures that a GN is in the communication range of at least one UAV. The cardinality of the set *Q* defines the maximum number of UAVs that can be used for each formed convex hull. In order to ensure connectivity among UAVs, we have considered using Algorithm 1, which verifies if each UAV has a path to the selected destination, which may be used as the control station. UAVs are assumed to have two main attributes: serving, when the UAV is used to serve GNs and to connect the network, and bridging, when it is solely being used to connect the serving UAVs.
**Algorithm 1** Construction of the connected UAV network.1:**Input**: $u_{dest}$, adjacency matrix2:**Result**: Connected UAV network3:**For each**u∈U4:   **IF** *u* is serving, and *u* is not bridging5:     $q^{curr}=q^{u}$; /*$q^{curr}\in Q$ is the current point towards the destination*/6:     **Until** not reachable (*u*,$u_{dest}$)  6.1Find the closest point $q^{\prime }$ ∈Q to $q^{u_{d}est}$, which is within distance D from $q^{curr}$  6.2**If**$q^{\prime }$ is not in use  6.2.1$\;q^{curr}=q^{\prime }$  6.2.2Find $u^{\prime }\in U$, which is not serving or bridging  6.2.3Set: $u^{\prime }$ to bridging  6.2.4$\;q^{u^{\prime }}=q^{curr}$  6.2.5Update the adjacency matrix

#### 4.2.2. Minimizing the Degree of Dissatisfaction of the Required Data Rate

Consider a set of transmission modes B comprising the possible bit rates $b_{i}$. We denote the transmission modes in use by a UAV and requested by a GN as $b_{i}^{u}$ and $b_{i}^{v}$, respectively. We define the degree of dissatisfaction as follows:

(8) $$\gamma^{v}=\left\{\begin{array}{cc} \frac{|b_{i}^{u}-b_{i}^{v}|}{b_{i}^{v}} & \mathrm{if}\;(b_{i}^{u}-b_{i}^{v})<0\\ 0 & \mathrm{Otherwise} \end{array}\right.$$

We consider that the use of a $b_{i}$ depends on the SNR. Usually, GNs experiencing a relatively low SNR will have their receiver interface tuned to a robust (with lower BER when compared with other modes under the same channel conditions) transmission mode with a lower data rate. On the other hand, if the SNR is relatively high, the receiver may be tuned to a transmission mode that offers a higher data rate. Essentially, from Equation (8), we measure the difference between the required data rate by the GN and the one that is delivered by the serving UAV given the current distance between them. As previously stated, LoS links are assumed between GNs and UAVs, and orthogonal channel codes are used to avoid interference between concurrent transmissions. Thus, if the distance between the UAV and GN is within the admissible distance for the required transmission mode $b_{i}^{v}$, then the GN can transmit at the required data rate, i.e., the degree of dissatisfaction is zero. In this work, we try to minimize the maximum dissatisfaction value as follows:

(9) $$min\;(max_{v\in V}\;\gamma^{v})$$

## 5. UAV Placement Based on NSGA-II

In this section, we present the terminologies used by NSGA-II [26] and the main genetic algorithm elements (individual or chromosome, fitness, selection, population, and genetic operators). The term solutions and individuals are interchangeably used along the remaining part of this paper.

NSGA-II is an elitist MOEA, which comprises two main procedures. One is the Pareto ranking procedure, which aims at sorting the population into different non-domination levels ($i_{rank}$) in ascending order. The lowest ranking level contains the best solution. In order to identify solutions of the first non-dominated front in a population of size *N*, each solution is compared with every other solution in the population to find if it is dominated. After all members of the first non-dominated front are found, they are discounted temporally so that the next non-dominated front could be found by repeating this first procedure. The other procedure is the diversity preservation, which is used to maintain a good spread of solutions in the obtained set of solutions. Members in each non-dominated front are assigned a value called *crowding distance* ($i_{distance}$). This value gives an estimate of the density of solutions surrounding a particular solution in the population. A solution with a smaller value of this distance measure is, in some sense, more crowded by other solutions. The crowded-comparison operator, denoted as ${≺}_{n}$, is used to distinguish the best solution during the selection process. It assumes that every individual *i* in the population has two attributes, $i_{rank}$ and $i_{distance}$. The partial order ${≺}_{n}$ is defined as:

(10) $$\begin{array}{c} i{≺}_{n}j\;\mathrm{if}\;\left(i_{rank}<j_{rank}\right)\\ \;\mathrm{or}\;(\left(i_{rank}=j_{rank}\right)\;\mathrm{and}\;\left(i_{distance}>j_{distance}\right)) \end{array}$$

That is, between two solutions with differing non-domination ranks, we prefer the solution with the lower (better) rank. Otherwise, if both solutions belong to the same front, then we prefer the solution that is located in a less crowded region.

Algorithm 2 shows the main loop of NSGA-II proposed by the authors in [26], where the call of the routines fast-non-dominated-sort ($R_{t}$) and crowding-distance-assignment ($F_{i}$) corresponds to the first and second procedure described above, respectively. $R_{t}$ is of size 2N formed by combining parent $S_{t}$ and offspring $Z_{t}$ populations. $F_{i}$ refers to the $i^{\mathrm{th}}$ front or level. The detailed explanation of the aforementioned procedures is also available in [26]. We describe the main loop of NSGA-II as follows:
**Algorithm 2** NSGA-II main loop.1:$R_{t}=S_{t}\cup Z_{t}$2:F = fast-non-dominated-sort ($R_{t}$)3:$S_{t+1}=\emptyset \;\mathrm{and}\;i=1$4:**Until**$|S_{t+1}|+F_{i}\leq N$  4.1.crowding-distance-assignment ($F_{i}$)  4.2.$S_{t+1}=S_{t+1}+F_{i}$  4.3.i=i+15:**Sort**($F_{i},{≺}_{n}$)6:$S_{t+1}=S_{t+1}\cup F_{i}\left[1:(N-\right|S_{t+1}\left|)\right]$7:$Z_{t+1}$ = make-new-population ($S_{t+1}$)8:t=t+1

Step 1. Combine parent and offspring population;
Step 2. $F=(F_{1},F_{2},\ldots )$; sort $R_{t}$ according to non-domination procedure;
Step 3. Initialize an empty set for the parent population $P_{t+1}=\emptyset$, and set a counter *i* to one;
Step 4. Until the parent population is filled;
  4.1. Calculate crowding-distance in $F_{i}$;
  4.2. Include the *i*th non-dominated front in the parent population;
  4.3. Check the next front for inclusion. The best solutions are in $F_{1}$. If the size of $F_{1}$ is smaller than *N*, we choose all the members of the set $F_{1}$ for the new population $S_{t+1}$. The remaining members of the population $S_{t+1}$ are chosen from the subsequent non-dominated front in the ascending order of their ranking, $\left(F_{2},F_{3},\ldots \right)$. Say that the set $F_{l}$ is the last non-dominated set beyond which no other set can be accommodated. In general, the count of solutions in all sets from $F_{1}$ to $F_{l}$ would be larger than the population size. In order to choose exactly *N* population members, we sort the solutions of the front $F_{l}$ using the crowded-comparison operator $\left({≺}_{n}\right)$ in descending order and choose the best solution needed to fill all population slots;
Step 5. Sort in descending order using ${≺}_{n}$;
Step 6. Choose the first $\left(N-|S_{t+1}|\right)$ elements of $F_{i}$;
Step 7. Use selection, crossover, and mutation to create a new population $Z_{t+1}$;
Step 8. Increment the generation counter.

### 5.1. Individual and Initial Population

**Figure 3:**
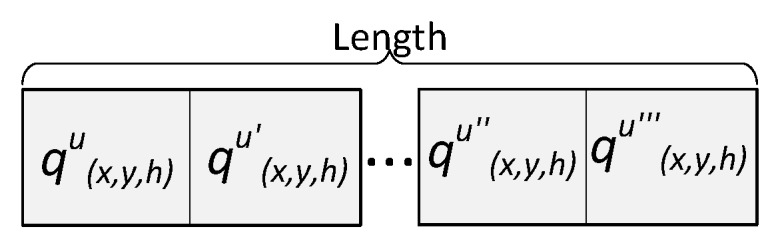
Individual.

An individual encodes a candidate solution to the problem. Our proposed individual stores the UAVs positions $q_{j}^{u}\in Q$ inside the discretized convex hull area $a^{\prime }$ for each deployed or serving UAV. The length of the individual (see Figure 3) represents the number of deployed UAVs or points used in *Q*; therefore, the length of one individual may differ from another in the population. If it is detected that some GNs are not covered in an individual, then the corresponding individual is considered as invalid, i.e., cannot be used in any step of the NSGA-II algorithm. Algorithm 1 ensures that all individuals are valid during the creation of the initial population.

In our implementation of NSGA-II, the initial population is a set of *N* randomly-generated valid individuals, i.e., random solutions to cover the target area.

### 5.2. Objective or Fitness Function

A fitness function decodes the solution represented by a chromosome and lets us know how far we are from the optimal/ideal solution if it is known. In MOEA, there will be a fitness function for each objective space. Equations (5) and (9) compute the fitness for the number of UAVs and degree of dissatisfaction, respectively. Values scored from both objective functions are used by NSGA-II to set the *i*th front.

### 5.3. Selection

The goal of selection procedure is to pick the best individuals for the next generation. We use binary tournament selection based on crowded-comparison operator ${≺}_{n}$, as described in Section 5.

### 5.4. Genetic Operators

Genetic operators are responsible for generating new solutions to populate the next generations. In the next sections, we present how they are performed.

#### 5.4.1. Crossover

**Figure 4:**
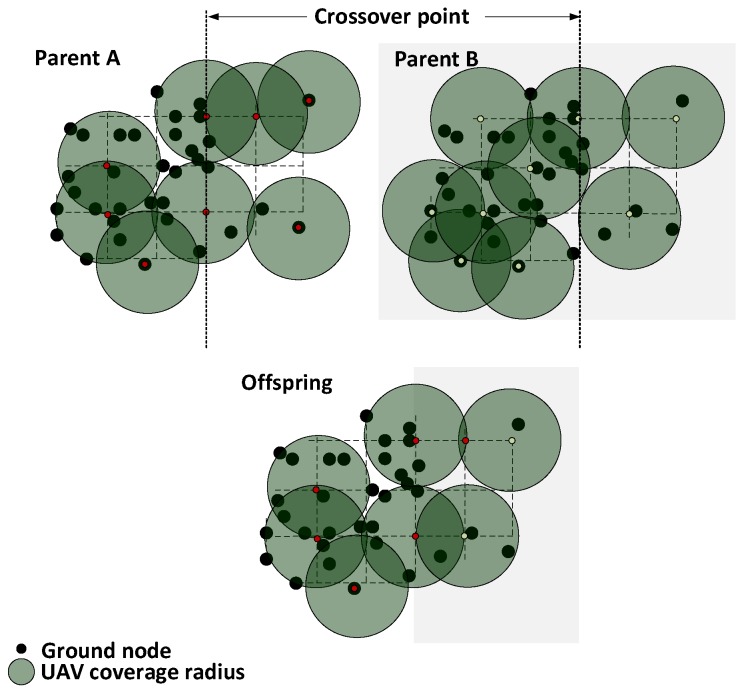
Crossover procedure.

Two parents are chosen to exchange their genes with a probability $p_{c}$. We rely on 2D representation of each parent (see Figure 4) to show how crossover is conducted. In this procedure, we find the midpoint in $a^{\prime }$ and draw a separation or cutting line to divide the area into two parts in each of the parents. The cutting line may be drawn diagonally in 45/−45 degrees or horizontally or vertically. Next, we remove all UAVs that are within a $\frac{1}{2}D$ distance radius along the cutting line within $a^{\prime }$. If the separation line is either diagonally or vertically drawn, the leftmost part of one parent is joined with the rightmost part of the other to form an offspring. On the other hand, if it is horizontally drawn, the uppermost and bottommost will be joined instead. There may be some uncovered GNs in the vicinity of the separation line, since we have removed some UAVs, which makes the resulting offspring an invalid individual. In this case, we repair the offspring by repeatedly choosing a random uncovered GN and place a UAV in a closest available point $q_{\left(x,y,h\right)}^{u}$ until all GNs are covered and the connectivity among UAVs is verified by Algorithm 1. UAVs that are not serving or bridging any GNs are removed.

#### 5.4.2. Mutation

**Figure 5:**
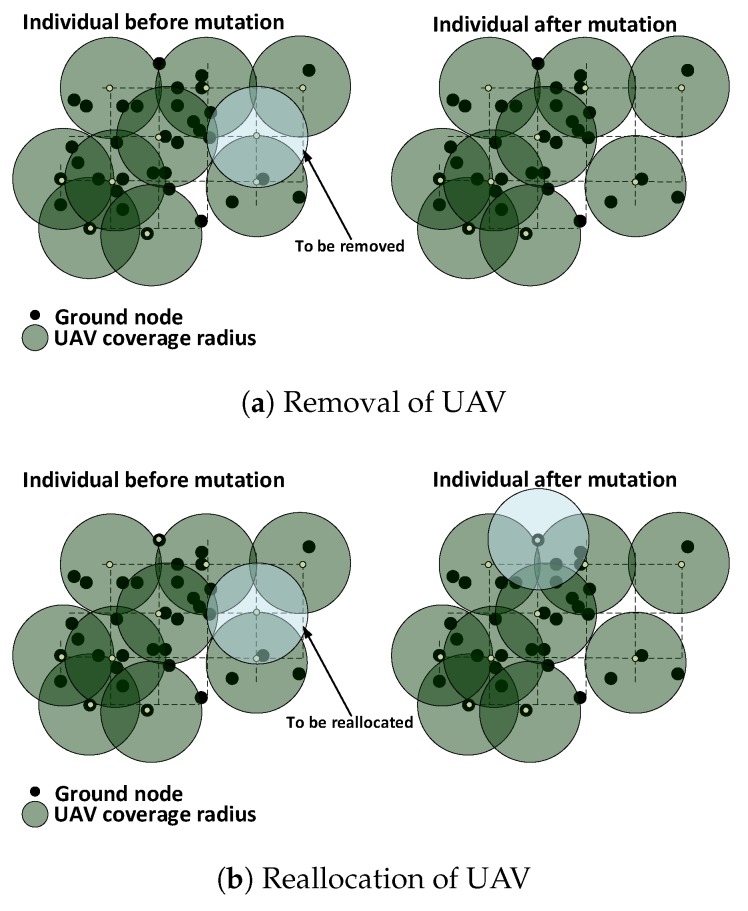
UAV removal and reallocation procedures during mutation.

For each individual, a UAV is randomly chosen based on a probability $p_{m}$. Next, either it is temporarilyremoved from the network or reallocated to a new available placement point with 50% chance for each procedure to be performed. If the above procedures fail to produce a valid individual, then the UAV is put back in its initial position. Figure 5a,b shows the removal and reallocation procedures, respectively.

### 5.5. Complexity

Determining the computational complexity of MOEA is difficult [27], since it depends on the specific problem setting. According to [26], the computational complexity of a faster algorithm such as NSGA-II is $O(MN^{2})$, where *M* is the number of objective functions and *N* is the population size. On the other hand, the authors in [27] have considered the computational complexity as $O(n_{g}MN^{2})$, where $n_{g}$ is the number of generations. Depending on the stopping criteria used, $n_{g}$ can have any complexity from constant to NP. It should be noted that when $n_{g}$ is not constant or otherwise limited, it is a function of the chromosome size. In this work, although we define a fixed cut-off value, as we will see later, the number of generations $n_{g}$ will be variable due to the variable length/size of the chromosome. The size of the chromosome is defined by the current number of deployed UAVs (candidate solution). In a population of size *N*, we may have many different solutions, which in turn makes it difficult to determine the computational complexity of our MOEA-NSGA-II implementation.

## 6. Simulation Results

In this section, we present the simulation results of our implementation of NSGA-II. We have two objective functions. The first one aims at reducing the cost in terms of the number of deployed UAVs used to service GNs, and the second one is intended to reduce the maximum dissatisfaction of GNs in terms of the required data rate. We have developed the algorithm in the C++ programming language. We assume that there will be a centralized entity (eventually located on one of the ground vehicles, or on the cloud) responsible for running the algorithm and delivering the configuration to the UAVs. The setup of the proposed scenarios, the MOEA termination criterion, and the dominated and non-dominated sets are presented in Section 6.1, Section 6.2 and Section 6.3, respectively.

### 6.1. Scenario Setup

We considered a network with 100 fixed GNs uniformly distributed in a rectangular area of size 5000 m × 5000 m. We set three different scenarios by varying the value of μ. This parameter is used to discretize the area inside the convex hull formed by the GNs. Different from our previous work [12] where UAVs were only allowed to fly at a fixed altitude, here, a UAV may fly at a given altitude *h* uniformly selected from the set H= {40, 80, 120} m. We assume that the transmit power among the nodes is fixed at 23 dBm. Previously, in Section 4, it was stated that potential UAV placement points will be identified within a convex hull formed by the GNs. The convex hull is found by the Graham scan algorithm [28] based on the GN deployment positions $q_{\left(x,y,0\right)}^{v}$.

Our scenarios consider a suburban environment and use log-distance path loss model for the signal attenuation. Table 2 shows all possible data rates and their corresponding minimum sensitivities at the receiver, i.e., $P_{r}\left(d\right)$. These values were used to compute the maximum achievable distance $D_{i}$ with α in Equation (1) set to 2.2 [23] and $d_{0}$ fixed at 1 m. We compute $P_{r}\left(d_{0}\right)$ using the free space propagation model, $P_{r}\left(d_{0}\right)=P_{t}G_{t}G_{r}{\left(c/4\pi d_{0}f\right)}^{2}$ [29], where $P_{t}$ is the transmit power, $G_{t}$ and $G_{r}$ are the transmitter and receiver antenna gains, respectively, and *c* and *f* are the speed of light and carrier frequency, respectively. We set $G_{t}$ = $G_{r}$ = 1 (0 dBm). Moreover, each data rate in Table 2 is considered to be using a different transmission mode.

For the set of UAV candidate position *Q*, we chose $D_{i}$ with the lowest minimum sensitivity and adjusted it by using the parameter μ to ensure that two UAVs positioned side by side can communicate with each other. As already stated, we assumed that there was a wireless communication technology between UAVs that was capable of efficiently relaying all the traffic from the GNs, never causing a bottleneck. The parameters that were common in different scenario are detailed in Table 3 as follows.

We have adjusted NSGA-II parameters such as the probability of crossover ($p_{c}$) and mutation ($p_{m}$) and the population size so that the algorithm did not prematurely converge or perform an excessive number of computations due to either low values of the probability of crossover or a high population size. The NSGA-II parameters are summarized in Table 4.

### 6.2. MOEA Termination Criterion

**Figure 6:**
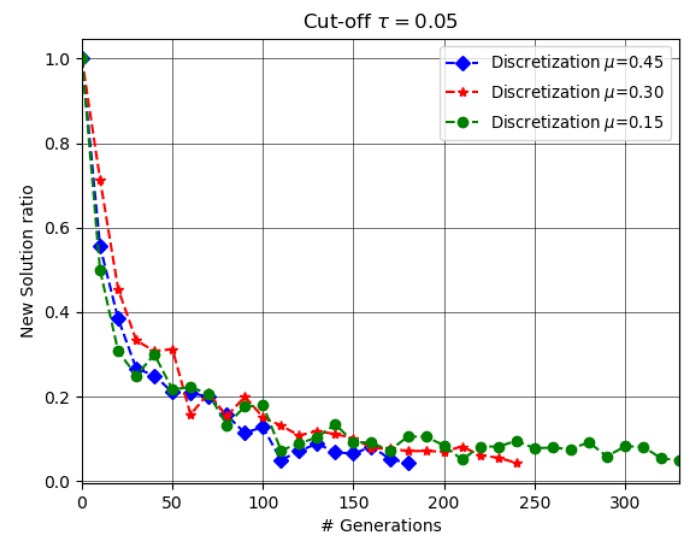
Ratio of new solutions.

The MOEA termination adopted in this work is similar to that used in [30], in the sense that we also maintained an external archive of non-dominated solutions obtained at some predefined steps at earlier generations, and it was subject to be updated some generations later. However, instead of computing the ratio of the number of solutions in the archive that were dominated by the new ones of the current generation and the ratio of the number of solutions that were also present in the new set of non-dominated solutions, we computed the ratio of new solutions, which were not present in both dominated and non-dominated sets of the archive, and we used it to define our stopping criterion. We used τ=0.05 as the cut-off value for the new solutions. However, the choice of the exact cut-off value may depend on the problem and may require some trial and error. Figure 6 shows the ratio of new solutions at every tenth generation (i.e., step = 10). The ratio was significantly high in the first generation when the algorithm was evolving and decreased with the generation, as new solutions were not frequent. We also observed that depending on μ, NSGA-II took a different number of generations to achieve the cut-off value. In fact, the value of μ affected the cardinality of *Q*, hence increasing or decreasing the search space, i.e., the higher the cardinality of *Q*, the higher was the number of generations to achieve the cut-off value. On the other hand, the lower the cardinality of *Q*, the lower was the number of generations to achieve the cut-off value. These results are shown in Table 5.

### 6.3. Dominated and Non-Dominated Sets

**Figure 7:**
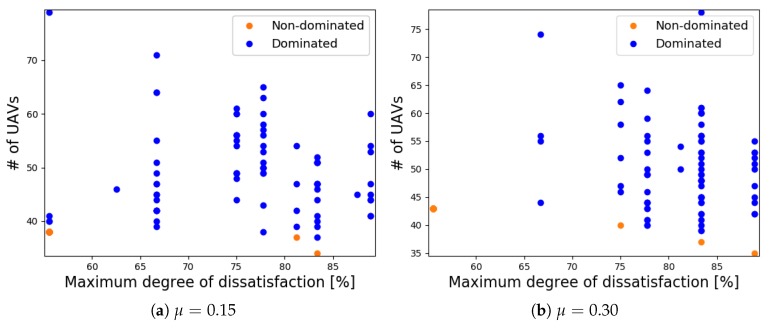

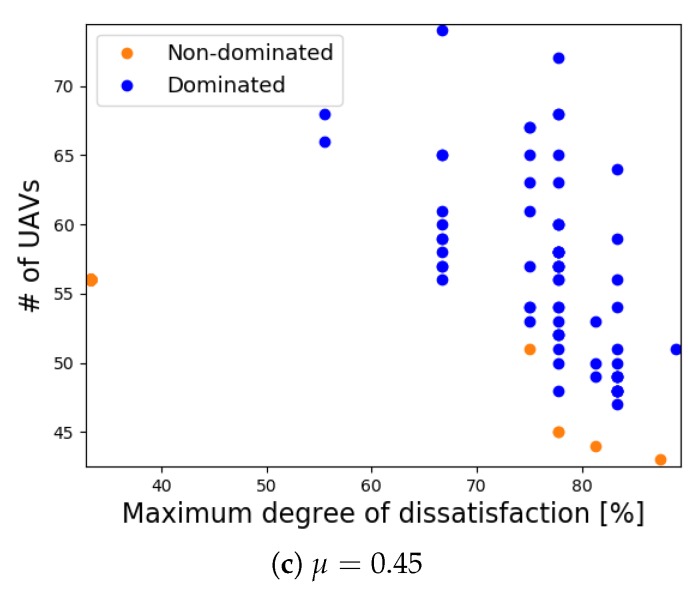
Trade-off between the number of UAVs and the degree of dissatisfaction of the GNs.

For each value of μ, all dominated and non-dominated solutions are presented in Figure 7. From each Pareto front set, we can clearly see the trade-off between the number of UAVs that are flying in the area and the degree of dissatisfaction of the GNs in terms of the required data rate, i.e., when few UAVs are deployed, a high degree of the maximum dissatisfaction was observed. On the other hand, when the number of UAVs increased, the degree of the maximum dissatisfaction decreased.

Table 6 presents the maximum and minimum number of UAVs and their respective degrees of dissatisfaction from the Pareto front set of each value of μ presented in Figure 7. These results show that the proposed algorithm can optimize the UAV placement given the requirement and the positions of the GNs in the target area.

## 7. Discussion

As shown above, varying μ affects the objective functions, though we have computed the convex hull to reduce the search space to some extent. However, this parameter may still reduce or increase the number of candidate points to place UAVs in the target area. The choice of μ depends on the requirement such as the area to be covered, the maximum transmission range, and also the number of available UAVs to cover the GNs to meet the QoS requirements.

In this work, varying the flying altitude would not significantly affect the objective functions given the order of magnitude between the communication range of omni-directional antennas considered in the simulations and the allowed flying altitudes from the set H, i.e., picking uniformly any value from the set H, there will be a slight difference between the covered areas.

The use of NSGA-II as an optimization tool allows us to produce a set of solutions that are better and spread as observed in our simulations results. It enables us to have options to select a solution according to the requirement of the application or problem at hand. For instance, if it is not acceptable that any GN communicates beyond 75% of the degree of dissatisfaction and there are no more than 60 available UAVs, then they can easily be configured with solutions that respect these requirements from our Pareto-optimal (non-dominated) set chosen from Figure 7.

The experimental results presented in the previous section are specific to the proposed scenarios and assumptions that were considered in our system model. In a realistic environment, one should take into account additional constraints such as the effect of interference, GN mobility, number of GNs to be covered, terrain conditions, etc.

- Interference: Nodes may be positioned within an acceptable distance for the required data rate, but may fail to achieve it due to interference caused by ongoing transmission of their neighboring nodes.
- GN mobility: Although the mobility is not considered in this work, it is worth mentioning that it would at least demand scheduling of periodic updates and computation of new solutions due to topology changes. As was previously mentioned, that is a challenging issue, namely because of the need to minimize temporary connectivity disruption due to UAV position changes.
- Number of GNs: UAVs have a limited capacity to service a certain number of GNs efficiently, and if this capacity is exceed, additional UAVs may be needed.
- Terrain conditions/ structure: UAVs may not fly at the desired altitude due to the existence of obstacles (e.g., trees, mountains, buildings, etc.), which may require the addition of more UAVs to maintain the connectivity among the nodes.

Algorithm 1 was used to ensure the connectivity of the network and produce valid solutions. We used the Breadth First Search (BFS) algorithm to check if there is a path to the destination. If a path is not found, it adds a new UAV to connect it, as explained in Section 4.2.1. This procedure is not optimized, which may conflict with the objective of minimizing the number of UAVs. However, it may eventually reduce the degree of dissatisfaction of the GNs.

## 8. Conclusions

This paper presents an optimized placement scheme for UAV access points providing network connectivity to GNs with differentiated data rate requirements. The goal of the proposed algorithm is to deploy as few as possible connected UAVs to cover and simultaneously satisfy the aforementioned requirements of the GNs. In order to attain this goal, we have mathematically formulated the problem and used an MOEA named NSGA-II to run the simulations. In order for NSGA-II to work, we proposed a chromosome structure, crossover scheme, and mutation procedure. Simulations were performed considering Wi-Fi (802.11g) technology, where GNs would request to turn to a given transmission mode within a set of available ones. Simulation results show that the algorithm optimizes the UAV placement given the requirements and positions of the GNs, considering the trade-off between the number of UAVs and the quality of the coverage.

In future work, we will consider additional constrains such as limited inter-UAV link capacity and interference. We will also consider joint topology and routing optimization.

## Figures and Tables

**Table 1:** Main characteristics of the related work on UAV placement optimization.

Reference	No. of UAVs	A2G Propag.Model	Antenna Type	Environment	A2A Communication
[2]	Single	LoS	-	-	-
[13]	Multiple	LoS	-	-	Yes
[14]	Multiple	LoS, NLoS	-	Urban	No
[15,16,17]	Multiple	LoS, NLoS	Isotropic	Urban	No
[18]	Single	LoS, NLoS	-	Suburban	No
[19]	Multiple	LoS, NLoS	Directional	Urban	No
[20]	Single	LoS, NLoS	-	Urban	No
[21]	Multiple	LoS	-	-	No
[22]	Multiple	LoS	Directional	-	Yes

**Table 2:** Maximum achievable distance of each transmission mode based on the minimum sensitivity of the receiver antenna.

Data Rate (Mbits/s)	Min. Sensitivity (dBm)	$D_{i}$ (m)
6	−82	892.24
9	−81	803.58
12	−79	651.81
18	−77	528.70
24	−74	386.23
36	−70	254.11
48	−66	167.19
54	−65	150.57

**Table 3:** Parameters in each scenario.

Parameters	Value
Transmit Power ($P_{t}$)	23 dBm
Antenna model	Omni-directional
Propagation model	Log-distance
Area A, ($X_{max}$× $Y_{max}$)	5000 m × 5000 m
No. of GNs	100
α	2.2
$d_{0}$	1 m
*c*	$3\times 10^{8}$ m/s
*f*	$2.412\times 10^{9}\;H_{z}$
$P_{r}\left(d_{0}\right)$	−47 dBm
μ	[0.15, 0.30, 0.45]
D	892.24 m

**Table 4:** NSGA-II setup parameters.

Parameters	Value
NSGA-II Population Size	80
NSGA-II $p_{c}$	0.9
NSGA-II $p_{m}$	0.6

**Table 5:** Number of generations achieved for cut-off τ=0.05 for each μ.

	μ=0.15	μ=0.30	μ=0.45
# of generations	330	240	180

**Table 6:** Maximum and minimum No. of UAVs for each scenario.

	Max. UAVs	Degree.Dissat(%)	Min. UAV	Degree. Dissat (%)
μ=0.15	38	55.55	34	83.33
μ=0.30	43	55.55	35	88.88
μ=0.45	56	33.33	43	87.50

## Abbreviations

The following abbreviations are used in this manuscript:

A2A	Air-to-Air
A2G	Air-to-Ground
BER	Bit Error Rate
BFS	Breadth First Search
BS	Base Station
D2D	Device-to-Device
FSPL	Free Space Path Loss
GN	Ground Node
GPS	Global Positioning System
IEEE	Institute of Electrical and Electronics Engineers
LoS	Line-of-Sight
MOEA	Multi-Objective Evolutionary Algorithm
MOP	Multi-objective Optimization Problem
NLoS	No Line-of-Sight
NSGA-II	Non-dominated Sorting Genetic Algorithm II
QoS	Quality of Service
PSO	Particle Swarm Optimization
RF	Radio Frequency
RSS	Received Signal Strength
SNR	Signal-to-Noise-Ratio
UAV	Unmanned Aerial Vehicles
UE	User Equipment
Wi-Fi	Wireless Fidelity
